# Structural characteristics in network control of molecular multiplex networks

**DOI:** 10.1371/journal.pone.0283768

**Published:** 2023-03-30

**Authors:** Cheng Yuan, Zu-Yu Qian, Jie Zhou, Shi-Ming Chen, Sen Nie

**Affiliations:** School of Electrical and Automation Engineering, East China Jiaotong University, Nanchang, Jiangxi, People’s Republic of China; Chongqing Three Gorges University, CHINA

## Abstract

Numerous real-world systems can be naturally modeled as multilayer networks, providing an efficient tool to characterize these complex systems. Although recent progress in understanding the controlling of synthetic multiplex networks, how to control real multilayer systems remains poorly understood. Here, we explore the controllability and energy requirement of molecular multiplex networks coupled by transcriptional regulatory network (TRN) and protein-protein interaction (PPI) network from the perspective of network structural characteristics. Our findings reveal that the driver nodes tend to avoid essential or pathogen-related genes. However, imposing external inputs on these essential or pathogen-related genes can remarkably reduce the energy cost, implying their crucial role in network control. Moreover, we find that the minimal driver nodes, as well as the energy required, are associated with disassortative coupling between TRN and PPI networks. Our results provide a comprehensive understanding of the roles of genes in biology and network control across several species.

## Introduction

Networks are prevalent in exploring the phenomenons and principles of our daily lives, e.g., traffic [1, 2], financial [3, 4], biological [5–8]and social systems [9, 10]. As the ultimate goal regarding the exploration of these systems is to drive them to desired states, numerous advances have been achieved in the field of network control [11–16]. Controllability as the first step of control, quantifies whether a given system can be driven from any initial state to any desired state within finite time with finite external inputs. Liu et al. [11] creatively combined structural controllability theory with complex networks and proposed a method to determine the minimal number of inputs (driver nodes) needed to fully control directed networks. Exact controllability is another framework used to analyze the controllability of complex networks with arbitrary structures and link weights [14]. Additionally, minimal inputs [17–20], the optimal control strategy [21, 22], the control energy [23–34], and the relationships between the structural characteristics of a network and controllability have been explored [35–40].

Biological systems, with large-scale and complicated interactions, can also provide graphs of whole cells and entire organisms. The study of biological systems based on network concepts has recently attracted much attention [41–44]. With the further goal of controlling systems, control theory has also been applied to biological networks to reveal the underlying mechanisms behind life processes [43–48]. Therefore, many network-based approaches have been developed to analyze the characteristics of biological networks, e.g., identifying disease genes [49] and drug-target interactions [50].

The functioning of many systems usually requires coupling between different types of networks [51–58]. Biological networks also function as consequences of the complex interactions between different molecular networks [41, 59–61]. For instance, proteins in a protein-protein interaction (PPI) network are translated from genes in a transcriptional regulatory network (TRN) [59]. Recently, Mahajan et al. [59] explored the interactions between the TRN and PPI networks of different species and revealed the impact of multiplex architectures on network robustness. They found that the functionally essential genes and proteins are situated in important parts of the multiplex networks.

Those coupled structures of multilayers not only influence the robustness of networks but also have an effect on other functions and abilities of molecular networks, e.g., controllability and control energy. In addition, our understanding of how those essential genes and proteins impact the network control remains a gap. In this study, based on the principles of control for single-layer biological networks, we examine the associations between the functional characteristics of genes and their roles in network control. We show that imposing external inputs on essential or pathogen-related genes can efficiently reduce the required control energy, even though the minimal driver node set tends to avoid these genes. Moreover, we find that a negative correlation between the TRN and PPI layers can simultaneously decrease the number of driver nodes and the required energy.

## Model

**Fig 1:**
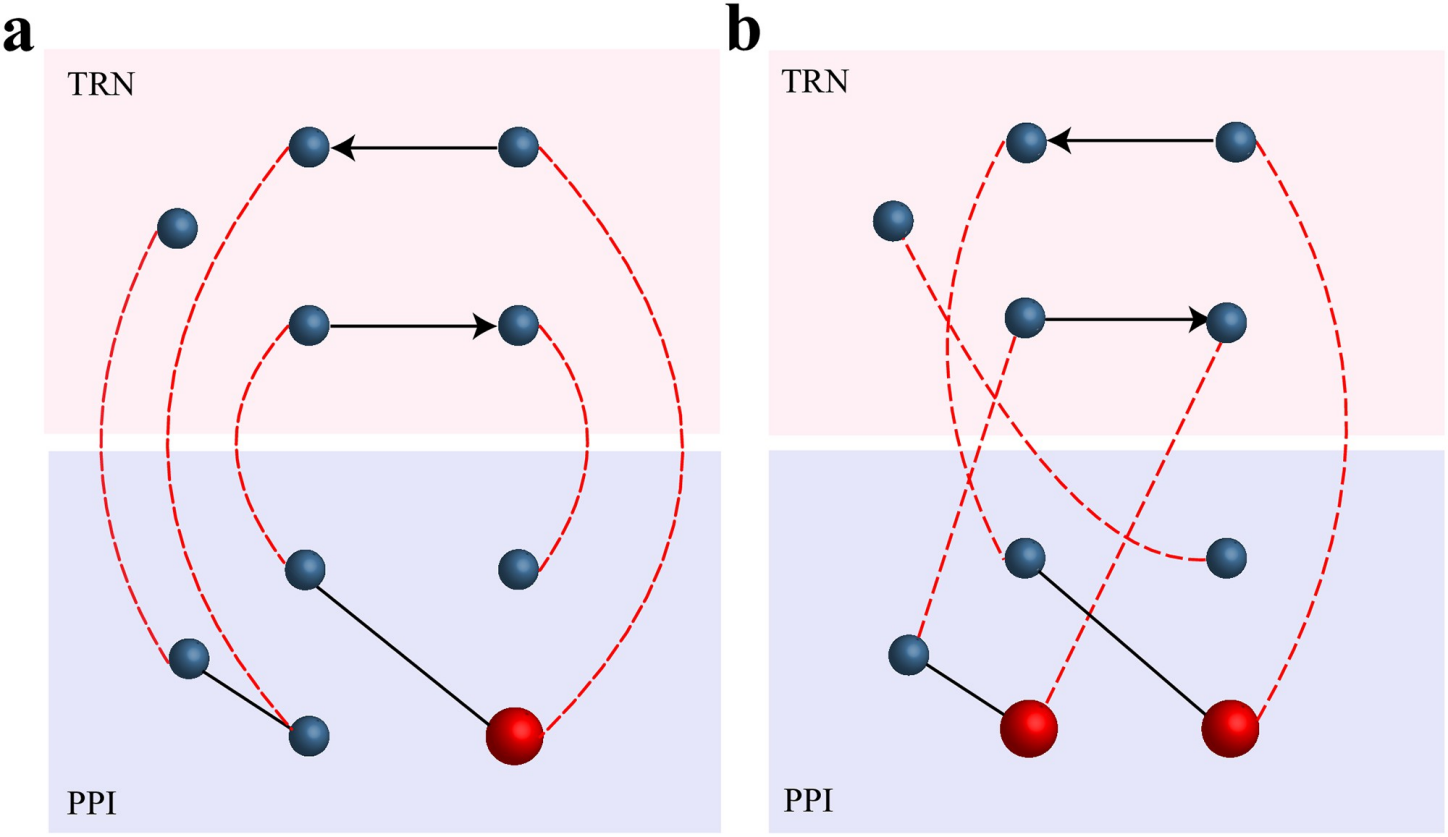
Schematic diagram of TRN-PPI multiplex networks with different interlayer couplings. The nodes in the two layers represent the genes and proteins. The red nodes are the minimal driver nodes that are imposed on the external inputs. The black directed lines represent the interactions between the transcription factors and their target genes in the TRN layer, and the black undirected lines represent the interactions between the proteins in the PPI layer. The red dashed lines represent the one-to-one interactions between the genes and their corresponding proteins. The interlayer couplings are different in **(a)** and **(b)**; thus, the minimal driver node sets vary.

Here, a transcriptional regulatory network (TRN) represents the interactions among transcription factors and their target genes. An edge in the TRN encodes the direct interactions between a transcription factor and its target genes. In a protein-protein interaction (PPI) network, each node represents a protein, and an undirected link denotes a physical or binding interaction. The coupling links between layers represent the interactions between the genes in the TRN and the proteins in the PPI, in which the proteins translated from genes can also regulate other genes. Edges in the TRN encode direct interactions between a transcription factor and its target genes, in which the transcription factors are considered as genes in our model. The couplings between the TRN and PPI layers form a one-to-one correspondence, i.e., a gene in the TRN is connected to a corresponding protein in the PPI layer [59]. A schematic diagram of the constructed multiplex networks is shown in Fig 1. The datasets are provided by Mahajan and Dar [59], who collected data from 7 species: *H. pylori* [62, 63], *M. tuberculosis* [64, 65], *E. coli* [66–68], *C. elegans* [68–70], *A. thaliana* [68, 69, 71], *M. musculus* [68, 69, 72], and *H. sapiens* [68, 69, 72]. Since TRN and PPI networks have different genome and proteome coverage levels, only the genes and proteins present in both layers are considered in our analysis.

Formally, the dynamics of each node in the TRN-PPI multiplex network are described by

$$\begin{array}{c} \dot{z}\left(t\right)=f\left(z,v,t\right) \end{array}$$ (1)

where ***z***(*t*) = (*z*_1_(*t*), *z*_2_(*t*), ⋯, *z*_*N*+ *N*_(*t*))^*T*^ denotes the states of the 2*N* nodes at time *t*. *N* represents the sizes of both the TRN and PPI network. ***f***(*) = [*f*_1_(*), *f*_2_(*), ⋯, *f*_*N*+ *N*_(*)]^*T*^ captures the nonlinear dynamics of each node, and ***v***(*t*) = [*v*_1_(*t*), *v*_2_(*t*), ⋯, *v*_*M*_(*t*)]^*T*^ denotes the external inputs imposed on the multiplex network. This model can be used to conceptualize the regulatory interactions between genes and proteins, in which some genes can be translated into proteins. For simplicity, assume that the system is at a fixed point z^*^, where ***f***(z^*^, v^*^, *t*) = 0, and ***x***(*t*) = ***z***(*t*) − z^*^, ***u***(*t*) = ***v***(*t*) − v^*^. Eq 1 can be linearized as [5]

$$\begin{array}{c} \dot{x}\left(t\right)=[\begin{array}{cc} A_{11} & A_{12}\\ A_{21} & A_{22} \end{array}]x\left(t\right)+Bu\left(t\right) \end{array}$$ (2)

where $[\begin{array}{cc} A_{11} & A_{12}\\ A_{21} & A_{22} \end{array}]\equiv \frac{\partial f}{\partial z}{|}_{z^{*},v^{*}}$ is the global adjacency matrix of the network and captures the interactions between the 2*N* nodes. ***A***_11_ (***A***_22_) describes the connections between the nodes within TRN (PPI), and ***A***_12_ (***A***_21_) captures the intraconnections between the TRN and PPI network. $B\equiv \frac{\partial f}{\partial v}{|}_{z^{*},v^{*}}$ represents how the external inputs are imposed on nodes. ***u***(*t*) is the external input.

Note that if the linear system in Eq 2 is locally controllable along a trajectory in the state space, then the corresponding nonlinear system in Eq 1 is also controllable along the same trajectory [5, 73]. In addition, the linear control predictions are consistent with the control of nonlinear dynamics [73]. Hereafter, to apply the controllability and control energy framework, we focus on the linear system in Eq 2.

## Methods

### Minimal driver nodes and control energy

A system is controllable if it can be driven from any initial state to any desired state with finite external inputs within finite time. External signals can be applied to nodes in the network, these nodes controlled by external signals are driver nodes, and the minimal number of driver nodes measures the controllability of a network. This is defined as *N*_D_, and $n_{\text{D}}=\frac{N_{\text{D}}}{N}$. According to the exact controllability framework [14], *N*_D_ can be calculated as

$$\begin{array}{c} N_{\text{D}}=\underset{i}{\text{max}}(\mu (\lambda_{i})) \end{array}$$ (3)

where *λ*_*i*_(*i* = 1, 2, ⋯, *N*) represent the eigenvalues of the adjacency matrix and *μ*(*λ*_*i*_) is the geometric multiplicity. Moreover, the minimal driver node set can be identified in *B* to satisfy the following equation:

$$\begin{array}{c} rank(A-\lambda^{M}I_{\text{N}},B)=N \end{array}$$ (4)

where *A* is the adjacency matrix of the network, *λ*^*M*^ refers to the eigenvalue obtained according to the maximum geometric multiplicity *μ*(*λ*^*M*^) and *I*_N_ is the identity matrix. It has been noted that the rank of matrix [*A* − *λ*^*M*^*I*_N_, *B*] is determined by the number of linearly independent rows. By performing an elementary column transformation on the matrix *A* − *λ*^*M*^*I*_N_, we can obtain these linearly dependent rows. Therefore, the external inputs described by *B* should be imposed on the rows to eliminate all linear relations and make the matrix [*A* − *λ*^*M*^*I*_N_, *B*] fully ranked, and the corresponding minimal driver node set can be identified [14]. This method based on exact controllability is applied to arbitrary networks.

On the basis of optimal control theory [74], the energy required to control the system is $E\left(t_{0},t_{f}\right)=\int_{t_{0}}^{t_{f}}{\left\|u\left(t\right)\right\|}^{2}dt$, where *t*_0_ is the initial time and *t*_*f*_ is the final time. If the initial state **x**_0_ = 0 at *t*_0_ = 0, the minimal control energy is

$$\begin{array}{c} E\left(t_{f}\right)=x_{t_{f}}^{T}W^{-1}\left(0,t_{f}\right)x_{t_{f}} \end{array}$$ (5)

where $W\left(0,t_{f}\right)=\int_{0}^{t_{f}}e^{At}BB^{T}e^{A^{T}t}dt$ is the Gramian matrix. As the control energy decays to a nonzero stationary value as the time *t* increases, the control energy discussed here is *E*(*t*_*f*_ → ∞), and the Gramian matrix is *W*(0, *t*_*f*_ → ∞). We set the elements as $A_{ii}=-\left(\delta +\sum_{j=1}^{N}A_{ij}\right)$, where *δ* = 0.25 to ensure the stability of the whole system [25]. This self-loop can be considered as one of the components which affect the state of the gene or protein in the regulatory process.

### Degree-degree correlation

The associativity between the TRN and PPI layers is measured by Pearson’s correlation coefficient *ρ* [75]

$$\begin{array}{c} \rho =cor(k_{\text{out}},K)\\ =\frac{\sum_{i=0}^{n}\left(k_{\text{out}}\left(i\right)-{\bar{k}}_{\text{out}}\right)\sum_{i=0}^{n}\left(K\left(i\right)-\bar{K}\right)}{\sqrt{\sum_{i=0}^{n}{\left(k_{\text{out}}\left(i\right)-{\bar{k}}_{\text{out}}\right)}^{2}}\sqrt{\sum_{i=0}^{n}{\left(K\left(i\right)-\bar{K}\right)}^{2}}} \end{array}$$ (6)

where *k*_out_ is the out-degree of the nodes in the TRN, *K* is the degree of the nodes in the PPI network and *n* is the number of nodes in each layer.

### Simulated annealing

To finely tune the degree-degree correlation coefficient *ρ* of the multiplex networks, we adopt the simulated annealing algorithm [59, 76], which is used to change the value of the degree-degree correlation coefficient *ρ* by shuffling gene labels [59].

1. Randomly shuffle the gene labels in the TRN, and keep the protein labels in the PPI network unchanged. Then, calculate the absolute difference between the shuffled and desired degree-degree correlations as $\Delta^{P}=\left|cor_{k,K}^{P}-cor_{k,K}^{D}\right|$, where $cor_{k,K}^{P}$ is the present correlation between the nodes with *k*_out_ degrees in the TRN and nodes with *K* degrees in the PPI. $cor_{k,K}^{D}$ is the desired correlation. Save the present gene labeling of the TRN.
2. Selecting *M* genes in the TRN network randomly (here we set *M* = 10), and shuffle their labels. Save the current labeling of the TRN as a new sample. Then, define the new difference as $\Delta^{*}=\left|cor_{k,K}^{*}-cor_{k,K}^{D}\right|$, where $cor_{k,K}^{*}$ is the degree-degree correlation of the present network after randomly shuffling 10 gene labels, and $cor_{k,K}^{D}$ is the desired correlation.
3. Calculate the difference Δ = Δ^*P*^ − Δ^*^, and accept the new labeling in step 2 with probability:
  $$p=\left\{\begin{array}{rr} 1, & \text{if}\;\Delta \;\geq 0;\\ e^{\frac{\Delta }{T}}, & \text{if}\;\Delta \;<0, \end{array}\right.$$
  where *T* = *T*_0_*e*^−*λL*^ is the temperature, *L* is the number of iterations and *λ* is the rate parameter. The parameters are set as *T*_0_ = 1, 000 and *λ* = 0.01 [59].
4. If Δ^*P*^ is smaller than the defined value (here we set the defined value as 0.01), then stop; otherwise, repeat the process from step 2.

## Results

### Driver node distributions in TRN-PPI multiplex networks

**Fig 2:**
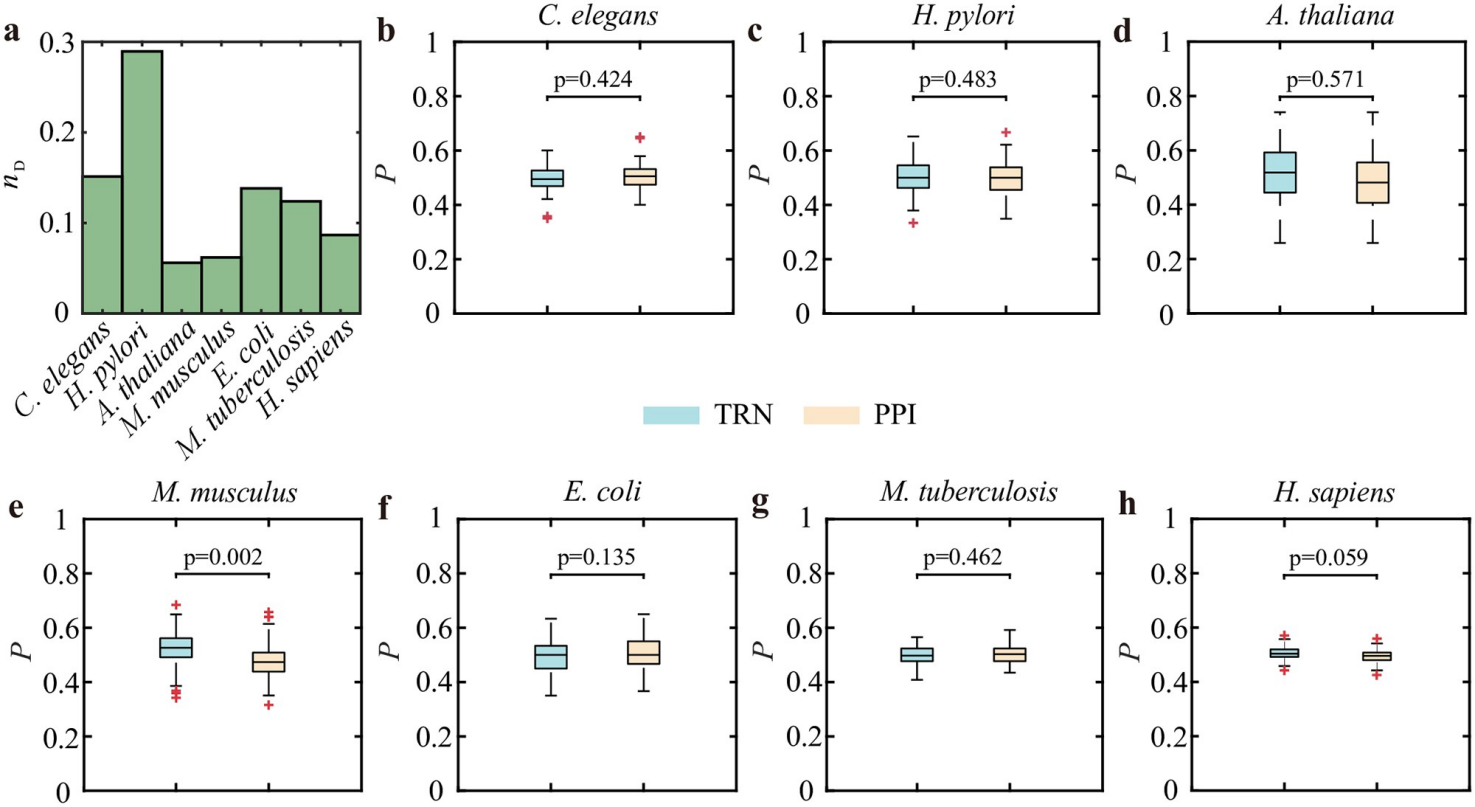
Controllability and driver node distributions of TRN-PPI multiplex networks. **(a)** The fractions of minimal driver nodes for seven species. **(b)-(h)** are the fractions of the minimal driver nodes in two layers for **(b)** *C. elegans*, **(c)** *H. pylori*, **(d)** *A. thaliana*, **(e)** *M. musculus*, **(f)** *E. coli*, **(g)** *M. tuberculosis*, and **(h)** *H. sapiens*. We obtain 100 minimal driver node sets and calculate the average values of the driver node fractions in the two layers. The p-value is calculated by the t-test.

To reflect the controllability of these TRN-PPI multilayer molecular networks, we first examine the minimal driver nodes *N*_D_ to achieve full control of the network for each species [14] (see Methods). The characteristics of the networks for each species are shown in Table 1. We find that most networks display lower *n*_D_ (∼0.15) than the *n*_D_ (∼0.3) of the *H. pylori* multiplex network and the values for some other real networks [14]. This indicates that we need to independently control approximately 15% nodes to fully control them. With its small average degree, *H. pylori* yields a high *n*_D_. This is consistent with previous findings that sparse networks are more difficult to control than dense networks [11]. Then, we examine the driver node distributions between the two layers, implying that these driver nodes do not have any particular preference regarding the two layers for most species (Fig 2(b)-2(h)). Strictly speaking, slightly more driver nodes are contained in the TRN than in the PPI layer for the *A. thaliana* and *M. musculus* species.

**Table 1:** The characteristics of the TRN-PPI multiplex networks for seven species.

species	*N*_ALL_	*L*_TRN_	*L*_PPI_	〈*k*〉_TRN_	〈*k*〉_PPI_
*C.elegens*	628	821	285	2.62	1.82
*H.pylori*	228	156	76	1.37	1.33
*A.thaliana*	482	385	398	1.60	3.30
*M.musculus*	920	1,269	518	2.76	2.25
*E.coli*	434	147	154	0.68	1.42
*M. tuberculosis*	1,542	958	946	1.24	2.45
*H.sapiens*	3,172	4,838	4,280	3.05	5.40

*N*_ALL_ is the number of all nodes in the network, *L*_TRN_ and *L*_PPI_ are the numbers of edges in the two subnetworks for the seven species, respectively. 〈*k*〉_TRN_ and 〈*k*〉_PPI_ represent the average degrees of the TRN and PPI, respectively.

### Roles of essential and pathogen-related genes in network controllability

**Fig 3:**
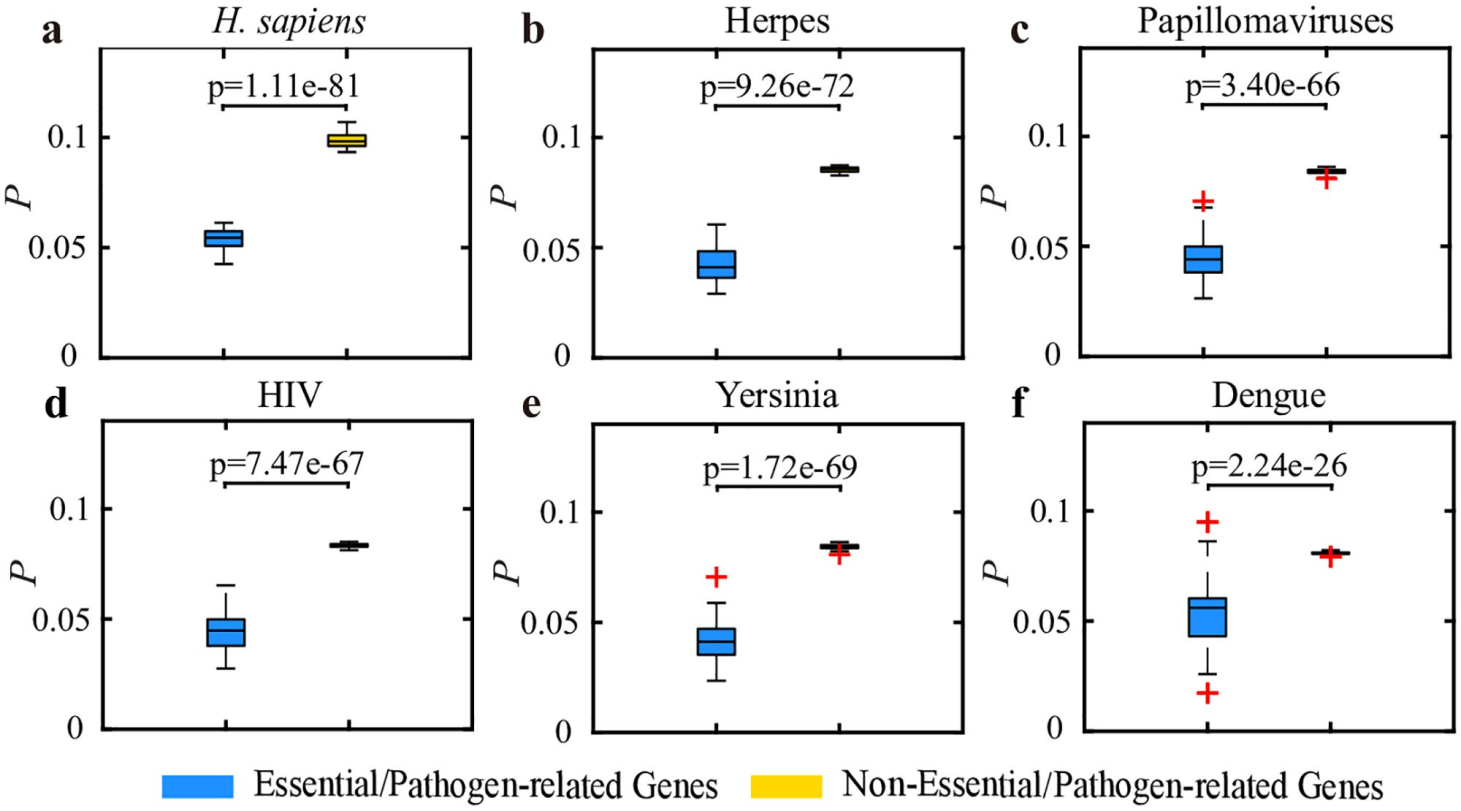
Distributions of the driver nodes in different gene categories. **(a)** *H. sapiens*, **(b)** Herpes, **(c)** Papillomaviruses, **(d)** HIV, **(e)** Yersinia, **(f)** Dengue. We obtain 100 minimal driver node sets and calculate the fractions of essential or pathogen-related genes among the driver node sets of all essential or pathogen-related genes (left, blue box), as well as the fractions of non-essential or nonpathogen-related genes among the driver node set of all non-essential or nonpathogen-related genes (right, yellow box), respectively. Then we obtain the average value. The red crosses represent the outliers, and the p-value is calculated by the t-test.

The essential and pathogen-related genes are critical for the survival and health status of an organism; thus, they convey particular topological characteristics in the corresponding multiplex networks [59]. Hence, we examine whether these essential or pathogen-related genes are associated with a prominent network control role. To achieve this, we adopt the gene categories in Ref. [59], where the essential genes for the human species are collected from the Online GEne Essentiality (OGEE) database [77, 78], and five human pathogens are collected from the publicly available database named HPIDB 3.0 [79, 80]. Then, we compare the fractions of essential or pathogen-related genes and the non-essential or nonpathogen-related genes are selected as the driver nodes. Fig 3 shows that the proportion of essential or pathogen-related genes selected as driver nodes is significantly lower than that of non-essential or nonpathogen-related genes (p-value<0.001), regardless of the considered species. It indicates that the driver nodes tend to avoid the essential or pathogen-related genes in TRN-PPI networks.

### Effect of interlayer coupling on network control

**Fig 4:**
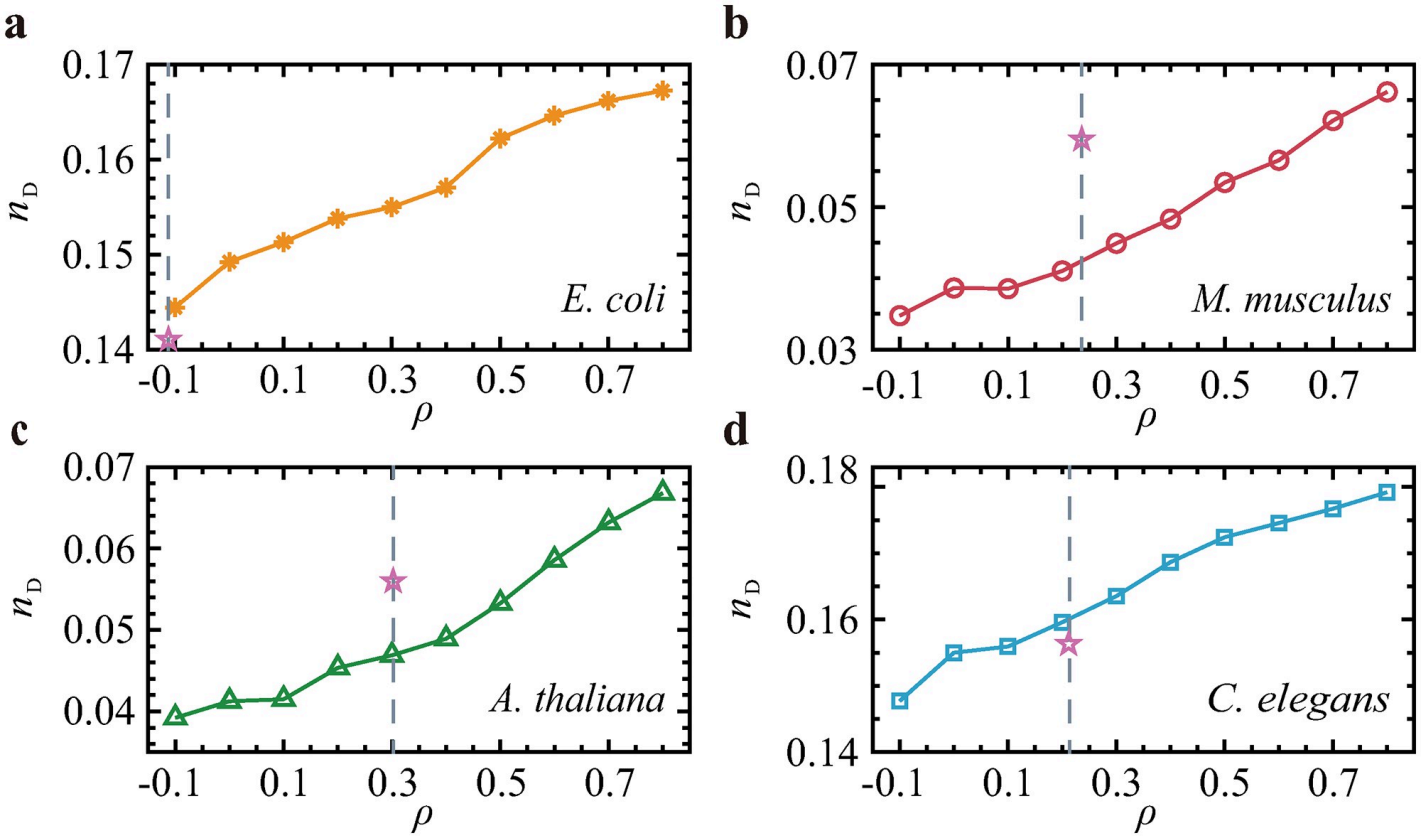
The minimal numbers of driver nodes as functions of the degree-degree correlations between the layers of TRN-PPI multiplex networks. **(a)** *E. coli*, **(b)** *M. musculus*, **(c)** *A. thaliana*, and **(d)** *C. elegans*. The gray dashed lines show the real values of the interlayer degree-degree correlations, and the purple pentagrams show the real values of *n*_D_ for the real connections between layers. Each data point is the mean of 100 independent realizations.

We further explore the impact of interlayer correlation on network controllability. Note that the interactions between the TRN and PPI network have already been established by their biological relationships. The purpose of our analysis is to reveal whether the underlying coupling pattern in reality can be partially explained in a network control manner. Therefore, we finely tune the correlation between the TRN and PPI layers through a simulated annealing algorithm [59, 76] (see Methods), finding that more driver nodes are required as the network transitions from disassortative to assortative (see Fig 4). We note that the real interlayer correlations for the four species are mostly positive [59]. We calculate the real interlayer correlations and the actual value of *n*_D_ and compare the actual value of *n*_D_ with the corresponding 100 random realizations. The results show that the actual value of *n*_D_ is close to the 100 random realizations.

**Fig 5:**
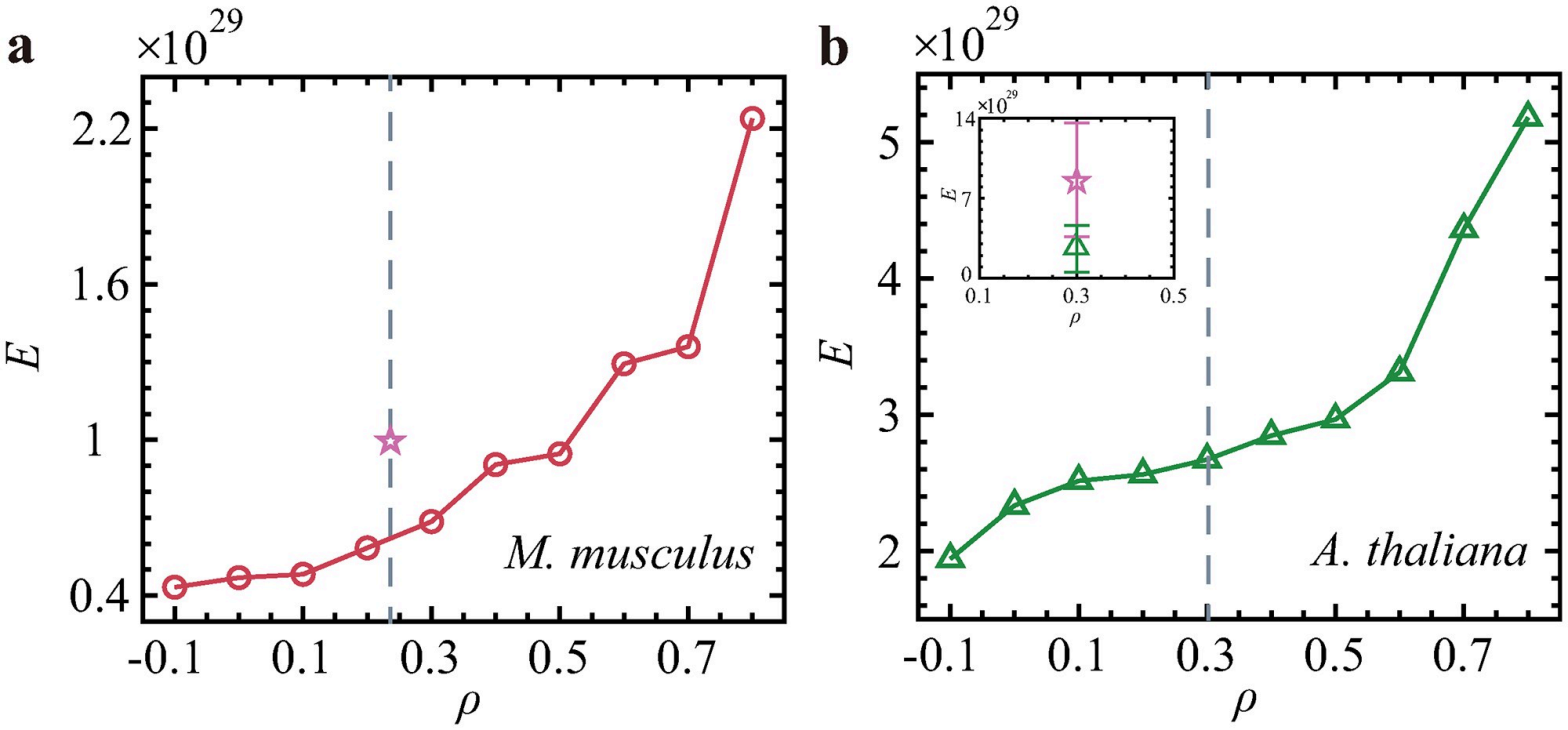
Control energy as functions of the degree-degree correlations between the layers of TRN-PPI multiplex networks. **(a)** *M. musculus*, and **(b)** *A. thaliana*. The initial state is *x*_0_ = [0, 0, ⋯, 0]^*T*^ and the final state is $x_{t_{f}}={\left[1,1,\ldots ,1\right]}^{T}$. The numbers of driver nodes for the two eukaryotes are 570 and 200, respectively. The gray dashed lines show the real values of the interlayer degree-degree correlations, and the purple pentagrams show the real values of *n*_D_ for the real connections between layers. The purple and green solid lines in the inset are used to represent the errors between the real value of *E* and the corresponding 100 random realizations, respectively. Each data point is the mean of 100 independent realizations.

We also assess how degree correlation determines the energy required for network control. We again finely tune the interlayer correlation by rewiring the links. To eliminate the impact of utilizing different driver node sets, all nodes are independently controlled. We find that the control energy in terms of degree correlation displays a trajectory similar to that observed in *n*_D_ (in Fig 4), i.e., multiplex networks with disassortative couplings between their layers are easier to control than those with assortative couplings (see Fig 5). The real values of *E* for the original connections between the layers are also calculated (see Fig 5). Though the values of *ρ* are the same for both the original networks and the simulated models, the real values of *n*_D_ and *E* are not the same as the results obtained by simulation. Since the topological structure of the network is changed in every independent realization, the driver nodes and control energy are all changed. The errors are positive and negative; this is allowable.

### Essential and pathogen-related genes are critical for the control energy

**Fig 6:**
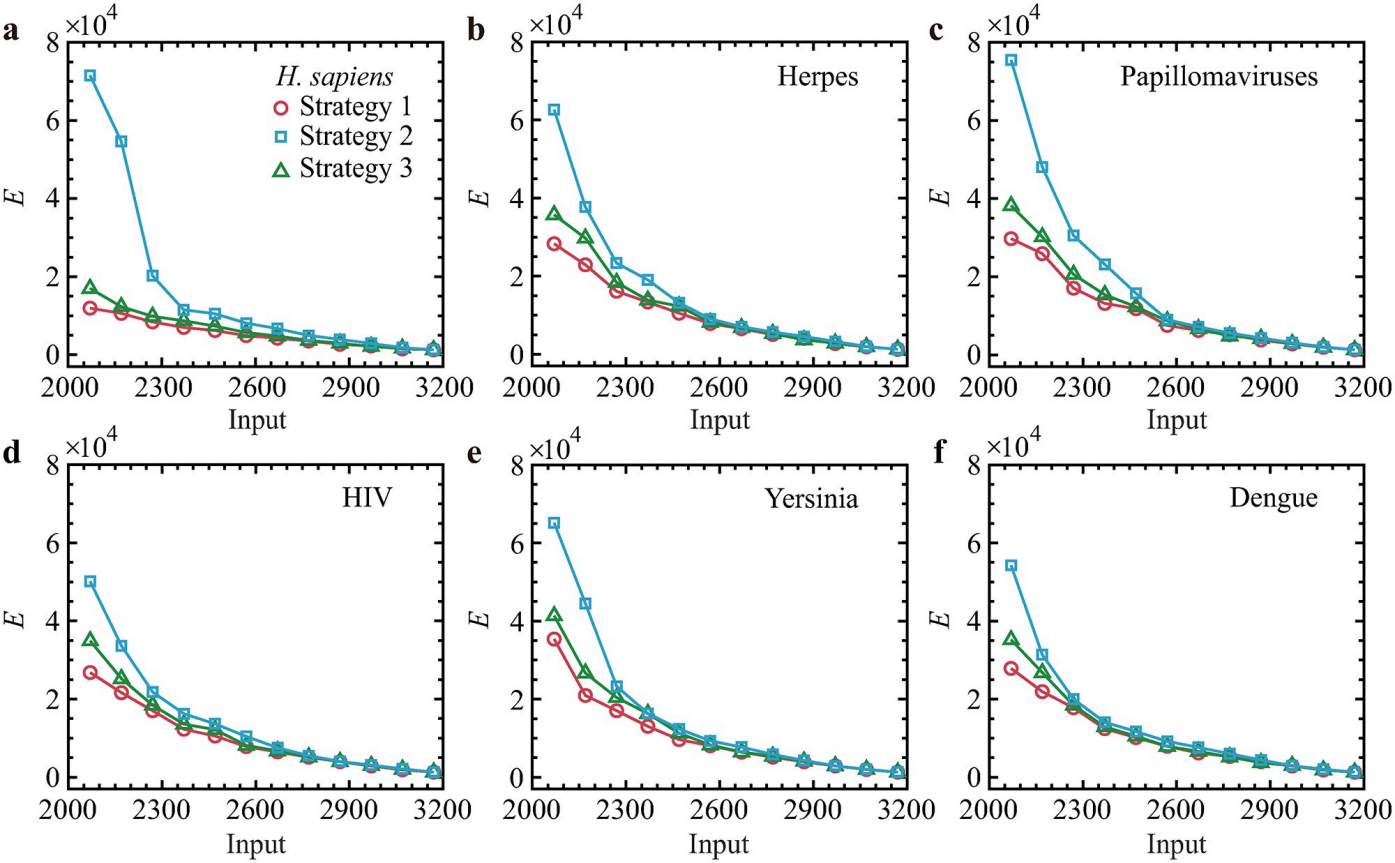
Control energy as functions of the number of driver nodes provided by three strategies. **(a)** *H. sapiens*. We choose all 1, 340 essential genes as driver nodes for *H. sapiens* and all pathogen-related genes as driver nodes for five different pathogens in **(b)-(f)**. The numbers of pathogen-related genes are 414 for **(b)**, 340 for **(c)**, 291 for **(d)**, 340 for **(e)**, and 116 for **(f)**. Each data point is the mean of 100 independent realizations.

Finally, we examine whether these essential genes can fill critical roles regarding the energy required for TRN-PPI multiplex network control. Subsequently, we propose three driver node selection strategies with the given *N*_D_. (1) All essential or pathogen-related genes are selected, where *N*_E_ are selected as driver nodes, and then the remaining *N*_D_ − *N*_E_ driver nodes are selected randomly. (2) *N*_D_ driver nodes are selected based on the in-degrees of all nodes in the TRN layer in descending order. (3) All *N*_D_ driver nodes are selected at random. Interestingly, we find that the control energies of these three strategies exhibit quite consistent implications: controlling those essential or pathogen-related genes yields the lowest energy required (see Fig 6).

To further explore the results, we find that the essential or pathogen-related genes are located in critical positions in the network structure. The control energy flows through the network by the control chain, which starts from a driver node and ends at a non-driver node along the shortest path between them [27]. The energy for controlling the non-driver nodes increases exponentially with the distance between the non-driver nodes and driver nodes. Thus, a shorter control chain leads to lower control energy.

Furthermore, the betweenness centrality (BC) is an index used to evaluate the importance of a nodal structure; its value is the fraction of shortest paths in the network going through the given node [27]: $BC_{i}=\sum_{i\neq s\neq t}\left(n_{st}^{i}\right)/g_{st}$. *g*_*st*_ is the number of all shortest paths from node *s* to node *t*, and $n_{st}^{i}$ is the number of shortest paths through node *i* among the *g*_*st*_ shortest paths from node *s* to node *t*. A node with a larger BC value has more paths between the node pairs going through it. Then, we find that the essential/pathogen-related genes are the nodes with larger BC values. The results in Table 2 show that the BC values of the essential/pathogen-related genes *B*_E_ are larger than those of the non-essential genes *B*_NE_ for most species. This indicates that the essential genes are the nodes that are more likely to be located in the middle positions of the paths between the driver nodes and non-driver nodes. Therefore, selecting these essential nodes as driver nodes reduces the length of the control chains, and the required energy becomes lower.

**Table 2:** The average betweenness centrality values of the essential genes (*B*_E_) and non-essential genes (*B*_NE_) for six species.

Species	*B*_E_(*10^−4^)	*B*_NE_(*10^−4^)
*H. sapiens*	7.26	10.60
*Herpes*	15.90	4.92
*Papillomaviruses*	17.80	5.05
*HIV*	14.40	6.30
*Yersinia*	15.60	5.65
*Dengue*	5.14	7.99

## Conclusion

Interactions between networks are ubiquitous in molecular networks. Here, we focus on multiplex networks consisting of transcriptional regulatory network (TRN) and protein-protein interaction (PPI) networks to explore their minimal driver nodes and the energy required for achieving full control. The results indicate that the driver nodes have no obvious preference for one layer over the other, and the driver nodes are more likely to avoid the essential or pathogen-related genes than other genes. In addition, the TRN-PPI networks with positive interlayer degree-degree correlations need more driver nodes and more energy to achieve full control. By comparing different driver node selection strategies, we find that the TRN-PPI networks require the lowest energy to reach the desired state by driving essential or pathogen-related genes. Our work bridges the gap between the structural characteristics of molecular multiplex networks and network control. It will be helpful for understanding the essential genes’ functions in biology and network control.

## Supporting information

S1 Data (ZIP)

S1 File (PDF)

## References

[1] Albert R, Barabási AL. Statistical mechanics of complex networks. Reviews of Modern Physics. 2002;74(1):47. doi: 10.1103/RevModPhys.74.47

[2] Newman ME. The structure and function of complex networks. SIAM Review. 2003;45(2):167–256. doi: 10.1137/S003614450342480

[3] Mantegna RN, Stanley HE. Introduction to econophysics: correlations and complexity in finance. Cambridge University Press; 1999.

[4] Jiang ZQ, Xie WJ, Zhou WX, Sornette D. Multifractal analysis of financial markets: a review. Reports on Progress in Physics. 2019;82(12):125901. doi: 10.1088/1361-6633/ab42fb 31505468

[5] Yan G, Vértes PE, Towlson EK, Chew YL, Walker DS, Schafer WR, et al. Network control principles predict neuron function in the Caenorhabditis elegans connectome. Nature. 2017;550(7677):519–523. doi: 10.1038/nature24056 29045391 PMC5710776

[6] Power JD, Cohen AL, Nelson SM, Wig GS, Barnes KA, Church JA, et al. Functional network organization of the human brain. Neuron. 2011;72(4):665–678. doi: 10.1016/j.neuron.2011.09.006 22099467 PMC3222858

[7] Jeong H, Mason SP, Barabási AL, Oltvai ZN. Lethality and centrality in protein networks. Nature. 2001;411(6833):41–42. doi: 10.1038/35075138 11333967

[8] Lee TI, Rinaldi NJ, Robert F, Odom DT, Bar-Joseph Z, Gerber GK, et al. Transcriptional regulatory networks in Saccharomyces cerevisiae. Science. 2002;298(5594):799–804. doi: 10.1126/science.1075090 12399584

[9] Deng C, Ye C, Wang L, Rong Z, Wang X. Peer pressure and incentive mechanisms in social networks. EPL (Europhysics Letters). 2018;121(1):18003. doi: 10.1209/0295-5075/121/18003

[10] Wang XW, Zhang HF, Nie S, Wang BH. Evolution of public cooperation with weighted and conditional strategies. Physica A: Statistical Mechanics and its Applications.2013;392(19):4668–4674. doi: 10.1016/j.physa.2013.05.020

[11] Liu YY, Slotine JJ, Barabási AL. Controllability of complex networks. Nature. 2011;473(7346):167–173. doi: 10.1038/nature10011 21562557

[12] Liu YY, Barabási AL. Control principles of complex systems. Reviews of Modern Physics. 2016;88(3):035006. doi: 10.1103/RevModPhys.88.035006

[13] Li A, Liu YY. Controlling network dynamics. Advances in Complex Systems. 2019;22(07n08):1950021.

[14] Yuan ZZ, Zhao C, Di ZR, Wang WX, Lai YC. Exact controllability of complex networks. Nature Communications. 2013;4(1):1–9. doi: 10.1038/ncomms3447 24025746 PMC3945876

[15] Liu LZ, Wang YH, Chen HG, Gao ZL. Synchronization control for discrete-time complex dynamical networks with dynamic links subsystem. Modern Physics Letters B. 2020;34(31):2050352. doi: 10.1142/S0217984920503522

[16] Gao ZL, Liu LZ, Wang YH, Gao PT, Li YF. Stabilization and synchronization control for complex dynamical networks with dynamic link subsystem. Information Sciences. 2022;609:1588–1600. doi: 10.1016/j.ins.2022.07.153

[17] Pósfai M, Liu YY, Slotine JJ, Barabási AL. Effect of correlations on network controllability. Scientific Reports. 2013;3(1):1–7. doi: 10.1038/srep01067 23323210 PMC3545232

[18] Xiang L, Chen F, Ren W, Chen G. Advances in network controllability. IEEE Circuits and Systems Magazine. 2019;19(2):8–32. doi: 10.1109/MCAS.2019.2909446

[19] Ramos G, Aguiar AP, Pequito S. An overview of structural systems theory. Automatica. 2022;140:110229. doi: 10.1016/j.automatica.2022.110229

[20] Gao LT, Zhao GS, Li GQ, Guo FH, Zeng F. Optimal target control of complex networks with selectable inputs. IEEE Transactions on Control of Network Systems. 2020;8(1):212–221. doi: 10.1109/TCNS.2020.3024318

[21] Gao J, Liu YY, D’souza RM, Barabási AL. Target control of complex networks. Nature Communications. 2014;5(1):1–8. doi: 10.1038/ncomms6415 25388503 PMC4243219

[22] Xiao YD, Lao SY, Hou LL, Bai L. Edge orientation for optimizing controllability of complex networks. Physical Review E. 2014;90(4):042804. doi: 10.1103/PhysRevE.90.042804 25375546

[23] Yan G, Ren J, Lai YC, Lai CH, Li B. Controlling complex networks: How much energy is needed? Physical Review Letters. 2012;108(21):218703. 23003312 10.1103/PhysRevLett.108.218703

[24] Sun J, Motter AE. Controllability transition and nonlocality in network control. Physical Review Letters. 2013;110(20):208701. doi: 10.1103/PhysRevLett.110.208701 25167459

[25] Yan G, Tsekenis G, Barzel B, Slotine JJ, Liu YY, Barabási AL. Spectrum of controlling and observing complex networks. Nature Physics. 2015;11(9):779–786. doi: 10.1038/nphys3422

[26] Nie S, Wang XW, Wang BH, Jiang LL. Effect of correlations on controllability transition in network control. Scientific Reports. 2016;6(1):1–9. doi: 10.1038/srep23952 27063294 PMC4827056

[27] Chen YZ, Wang LZ, Wang WX, Lai YC. Energy scaling and reduction in controlling complex networks. Royal Society Open Science. 2016;3(4):160064. doi: 10.1098/rsos.160064 27152220 PMC4852643

[28] Lindmark G, Altafini C. Minimum energy control for complex networks. Scientific Reports. 2018;8(1):1–14. doi: 10.1038/s41598-018-21398-7 29453421 PMC5816648

[29] Wang LZ, Chen YZ, Wang WX, Lai YC. Physical controllability of complex networks. Scientific Reports. 2017;7(1):1–14. 28074900 10.1038/srep40198 PMC5225471

[30] Nie S, Stanley HE, Chen SM, Wang BH, Wang XW. Control energy of complex networks towards distinct mixture states. Scientific Reports. 2018;8(1):1–8. doi: 10.1038/s41598-018-29207-x 30022118 PMC6052030

[31] Pasqualetti F, Zampieri S, Bullo F. Controllability metrics, limitations and algorithms for complex networks. IEEE Transactions on Control of Network Systems. 2014;1(1):40–52. doi: 10.1109/TCNS.2014.2310254

[32] Klickstein I, Sorrentino F. Selecting energy efficient inputs using graph structure. International Journal of Control. 2022;1(1):1–13. doi: 10.1080/00207179.2021.2022218

[33] Meng T, Duan GP, Li AM, Wang L. Control energy scaling for target control of complex networks. Chaos, Solitons & Fractals. 2023;167:112986. doi: 10.1016/j.chaos.2022.112986

[34] Chen H, Yong EH. Optimizing target nodes selection for the control energy of directed complex networks. Scientific Reports. 2020;10(1):18112. doi: 10.1038/s41598-020-75101-w 33093576 PMC7581767

[35] Nepusz T, Vicsek T. Controlling edge dynamics in complex networks. Nature Physics. 2012;8(7):568–573. doi: 10.1038/nphys2327

[36] Jia T, Liu YY, Csóka E, Pósfai M, Slotine JJ, Barabási AL. Emergence of bimodality in controlling complex networks. Nature Communications. 2013;4(1):1–6. doi: 10.1038/ncomms3002 23774965

[37] Zhao C, Wang WX, Liu YY, Slotine JJ. Intrinsic dynamics induce global symmetry in network controllability. Scientific Reports. 2015;5(1):1–5. doi: 10.1038/srep08422 25672476 PMC4325315

[38] Wang XW, Nie S, Wang WX, Wang BH. Controlling complex networks with conformity behavior. EPL (Europhysics Letters). 2015;111(6):68004. doi: 10.1209/0295-5075/111/68004

[39] Liu XM, Li DQ, Ma MQ, Szymanski BK, Stanley HE, Gao JX. Network resilience. Physics Reports. 2022;971:1–108. doi: 10.1016/j.physrep.2022.04.002

[40] Montanari AN, Duan C, Aguirre LA, Motter AE. Functional observability and target state estimation in large-scale networks. Proceedings of the National Academy of Sciences. 2022;119(1):e2113750119. doi: 10.1073/pnas.2113750119 34969842 PMC8740740

[41] Barabási AL, Oltvai ZN. Network biology: understanding the cell’s functional organization. Nature Reviews Genetics. 2004;5(2):101–113. doi: 10.1038/nrg1272 14735121

[42] Liu C, Ma Y, Zhao J, Nussinov R, Zhang YC, Cheng F, et al. Computational network biology: data, models, and applications. Physics Reports. 2020;846:1–66. doi: 10.1016/j.physrep.2019.12.004

[43] Vinayagam A, Gibson TE, Lee HJ, Yilmazel B, Roesel C, Hu Y, et al. Controllability analysis of the directed human protein interaction network identifies disease genes and drug targets. Proceedings of the National Academy of Sciences.2016;113(18):4976–4981. doi: 10.1073/pnas.1603992113 27091990 PMC4983807

[44] Li M, Gao H, Wang J, Wu FX. Control principles for complex biological networks. Briefings in bioinformatics. 2019;20(6):2253–2266. doi: 10.1093/bib/bby088 30239577

[45] Wuchty S. Controllability in protein interaction networks. Proceedings of the National Academy of Sciences.2014;111(19):7156–7160. doi: 10.1073/pnas.1311231111 24778220 PMC4024882

[46] Kanhaiya K, Czeizler E, Gratie C, Petre I. Controlling directed protein interaction networks in cancer. Scientific reports. 2017;7(1):1–12. doi: 10.1038/s41598-017-10491-y 28871116 PMC5583175

[47] Guo WF, Zhang SW, Feng YH, Liang J, Zeng T, Chen LN. Network controllability-based algorithm to target personalized driver genes for discovering combinatorial drugs of individual patients. Nucleic Acids Research. 2021;49(7):e37. doi: 10.1093/nar/gkaa1272 33434272 PMC8053130

[48] Zhang T, Zhang SW, Li Y. Identifying driver genes for individual patients through inductive matrix completion. Bioinformatics. 2021;37(23):4477–4484. doi: 10.1093/bioinformatics/btab477 34175939

[49] Zhang XF, Ou-Yang L, Zhu Y, Wu MY, Dai DQ. Determining minimum set of driver nodes in protein-protein interaction networks. BMC bioinformatics. 2015;16(1):1–13. doi: 10.1186/s12859-015-0591-3 25947063 PMC4428234

[50] Zhao T, Hu Y, Valsdottir LR, Zang T, Peng J. Identifying drug–target interactions based on graph convolutional network and deep neural network. Briefings in bioinformatics. 2021;22(2):2141–2150. doi: 10.1093/bib/bbaa044 32367110

[51] Yuan Z, Zhao C, Wang WX, Di Z, Lai YC. Exact controllability of multiplex networks. New Journal of Physics. 2014;16(10):103036. doi: 10.1088/1367-2630/16/10/103036

[52] Nie S, Wang X, Wang B. Effect of degree correlation on exact controllability of multiplex networks. Physica A: Statistical Mechanics and its Applications.2015;436:98–102. doi: 10.1016/j.physa.2015.05.038

[53] Lee KM, Min B, Goh KI. Towards real-world complexity: an introduction to multiplex networks. The European Physical Journal B. 2015;88(2):1–20. doi: 10.1140/epjb/e2015-50742-1

[54] Battiston F, Nicosia V, Latora V. Structural measures for multiplex networks. Physical Review E. 2014;89(3):032804. doi: 10.1103/PhysRevE.89.032804 24730896

[55] Zhu P, Wang X, Li S, Guo Y, Wang Z. Investigation of epidemic spreading process on multiplex networks by incorporating fatal properties. Applied Mathematics and Computation. 2019;359:512–524. doi: 10.1016/j.amc.2019.02.049 32287502 PMC7112296

[56] Solé-Ribalta A, Gómez S, Arenas A. Congestion induced by the structure of multiplex networks. Physical Review Letters. 2016;116(10):108701. doi: 10.1103/PhysRevLett.116.108701 27015514

[57] Mucha PJ, Richardson T, Macon K, Porter MA, Onnela JP. Community structure in time-dependent, multiscale, and multiplex networks. Science. 2010;328(5980):876–878. doi: 10.1126/science.1184819 20466926

[58] Cozzo E, Banos RA, Meloni S, Moreno Y. Contact-based social contagion in multiplex networks. Physical Review E. 2013;88(5):050801. doi: 10.1103/PhysRevE.88.050801 24329202

[59] Mahajan T, Dar RD. Internetwork connectivity of molecular networks across species of life. Scientific Reports. 2021;11(1):1–15. doi: 10.1038/s41598-020-80745-9 33441907 PMC7806680

[60] Maniatis T, Reed R. An extensive network of coupling among gene expression machines. Nature. 2002;416(6880):499–506. doi: 10.1038/416499a 11932736

[61] Yeger-Lotem E, Sattath S, Kashtan N, Itzkovitz S, Milo R, Pinter RY, et al. Network motifs in integrated cellular networks of transcription–regulation and protein–protein interaction. Proceedings of the National Academy of Sciences.2004;101(16):5934–5939. doi: 10.1073/pnas.0306752101 15079056 PMC395901

[62] Danielli A, Amore G, Scarlato V. Built shallow to maintain homeostasis and persistent infection: insight into the transcriptional regulatory network of the gastric human pathogen Helicobacter pylori. PLoS Pathog. 2010;6(6):e1000938. doi: 10.1371/journal.ppat.1000938 20548942 PMC2883586

[63] Häuser R, Ceol A, Rajagopala SV, Mosca R, Siszler G, Wermke N, et al. A second-generation protein–protein interaction network of Helicobacter pylori. Molecular & Cellular Proteomics. 2014;13(5):1318–1329. doi: 10.1074/mcp.O113.033571 24627523 PMC4014287

[64] Sanz J, Navarro J, Arbués A, Martín C, Marijuán PC, Moreno Y. The transcriptional regulatory network of Mycobacterium tuberculosis. PloS One. 2011;6(7):e22178. doi: 10.1371/journal.pone.0022178 21818301 PMC3139605

[65] Wang Y, Cui T, Zhang C, Yang M, Huang Y, Li W, et al. Global protein- protein interaction network in the human pathogen Mycobacterium tuberculosis H37Rv. Journal of Proteome Research. 2010;9(12):6665–6677. doi: 10.1021/pr100808n 20973567

[66] Salgado H, Martínez-Flores I, Bustamante VH, Alquicira-Hernández K, García-Sotelo JS, García-Alonso D, et al. Using RegulonDB, the Escherichia coli K-12 Gene Regulatory Transcriptional Network Database. Current Protocols in Bioinformatics. 2018;61(1):1–32. doi: 10.1002/cpbi.43 30040192 PMC6060643

[67] Rajagopala SV, Sikorski P, Kumar A, Mosca R, Vlasblom J, Arnold R, et al. The binary protein-protein interaction landscape of Escherichia coli. Nature Biotechnology. 2014;32(3):285–290. doi: 10.1038/nbt.2831 24561554 PMC4123855

[68] Das J, Yu H. HINT: High-quality protein interactomes and their applications in understanding human disease. BMC Systems Biology. 2012;6(1):1–12. doi: 10.1186/1752-0509-6-92 22846459 PMC3483187

[69] Chatr-Aryamontri A, Oughtred R, Boucher L, Rust J, Chang C, Kolas NK, et al. The BioGRID interaction database: 2017 update. Nucleic Acids Research. 2017;45(D1):D369–D379. doi: 10.1093/nar/gkw1102 27980099 PMC5210573

[70] Fuxman Bass JI, Pons C, Kozlowski L, Reece-Hoyes JS, Shrestha S, Holdorf AD, et al. A gene-centered C. elegans protein–DNA interaction network provides a framework for functional predictions. Molecular Systems Biology. 2016;12(10):884. doi: 10.15252/msb.20167131 27777270 PMC5081483

[71] Jin J, He K, Tang X, Li Z, Lv L, Zhao Y, et al. An Arabidopsis transcriptional regulatory map reveals distinct functional and evolutionary features of novel transcription factors. Molecular Biology and Evolution. 2015;32(7):1767–1773. doi: 10.1093/molbev/msv058 25750178 PMC4476157

[72] Han H, Cho JW, Lee S, Yun A, Kim H, Bae D, et al. TRRUST v2: an expanded reference database of human and mouse transcriptional regulatory interactions. Nucleic Acids Research. 2018;46(D1):D380–D386. doi: 10.1093/nar/gkx1013 29087512 PMC5753191

[73] Coron JM. Control and nonlinearity. American Mathematical Soc.; 2007.

[74] Rugh WJ. Linear system theory. Prentice-Hall, Inc.; 1996.

[75] Foster JG, Foster DV, Grassberger P, Paczuski M. Edge direction and the structure of networks. Proceedings of the National Academy of Sciences.2010;107(24):10815–10820. doi: 10.1073/pnas.0912671107 20505119 PMC2890716

[76] Kirkpatrick S, Gelatt CD, Vecchi MP. Optimization by simulated annealing. Science. 1983;220(4598):671–680. doi: 10.1126/science.220.4598.671 17813860

[77] Chen WH, Lu G, Chen X, Zhao XM, Bork P. OGEE v2: an update of the online gene essentiality database with special focus on differentially essential genes in human cancer cell lines. Nucleic Acids Research. 2016;gkw1013. doi: 10.1093/nar/gkw1013 27799467 PMC5210522

[78] Chen WH, Minguez P, Lercher MJ, Bork P. OGEE: an online gene essentiality database. Nucleic Acids Research. 2012;40(D1):D901–D906. doi: 10.1093/nar/gkr986 22075992 PMC3245054

[79] Kumar R, Nanduri B. HPIDB -a unified resource for host-pathogen interactions. BMC Bioinformatics. 2010;11(6):1–6. doi: 10.1186/1471-2105-11-S6-S16 20946599 PMC3026363

[80] Ammari MG, Gresham CR, McCarthy FM, Nanduri B. HPIDB 2.0: a curated database for host–pathogen interactions. Database. 2016;2016. doi: 10.1093/database/baw103 27374121 PMC4930832

