# Supplementary material for: Structural characteristics in network control of molecular multiplex networks
Source: PLoS One. 2023 Mar 30;18(3):e0283768. doi: 10.1371/journal.pone.0283768 (PMC10062666; doi:10.1371/journal.pone.0283768)
Supplement: S1 File — (PDF) [file pone.0283768.s002.pdf]

Data:

TRN314.csv
Title: TRN layer of the C. elegans species
Legend: This file is the TRN layer of the C. elegans species

PPI314.csv
Title: PPI layer of the C. elegans species
Legend: This file is the PPI layer of the C. elegans species

TRN114.csv
Title: TRN layer of the H. pylori species
Legend: This file is the TRN layer of the H. pylori species

PPI114.csv
Title: PPI layer of the H. pylori species
Legend: This file is the PPI layer of the H. pylori species

TRN241.csv
Title: TRN layer of the A. thaliana species
Legend: This file is the TRN layer of the A. thaliana species

PPI241.csv
Title: PPI layer of the A. thaliana species
Legend: This file is the PPI layer of the A. thaliana species

TRN460.csv
Title: TRN layer of the M. musculus species
Legend: This file is the TRN layer of the M. musculus species

PPI460.csv
Title: PPI layer of the M. musculus species
Legend: This file is the PPI layer of the M. musculus species

TRN217.csv
Title: TRN layer of the E.coli species
Legend: This file is the TRN layer of the E.coli species

PPI217.csv
Title: PPI layer of the E.coli species
Legend: This file is the PPI layer of the E.coli species

TRN771.csv
Title: TRN layer of the M. tuberculosis species
Legend: This file is the TRN layer of the M. tuberculosis species

PPI771.csv
Title: PPI layer of the M. tuberculosis species
Legend: This file is the PPI layer of the M. tuberculosis species

TRN1586.csv
Title: TRN layer of the H. sapiens species
Legend: This file is the TRN layer of the H. sapiens species

PPI1586.csv
Title: PPI layer of the H. sapiens species
Legend: This file is the PPI layer of the H. sapiens species
